# Increased Risk of Cutaneous Melanoma Associated with *p53* Arg72Pro Polymorphism

**DOI:** 10.1371/journal.pone.0118112

**Published:** 2015-03-16

**Authors:** Peiliang Geng, Yunmei Liao, Zhihua Ruan, Houjie Liang

**Affiliations:** Department of Oncology and Southwest Cancer Center, Southwest Hospital, Third Military Medical University, China; Federal University of Pelotas, BRAZIL

## Abstract

**Objective:**

The objective of this study was to test the hypothesis that *p53* Arg72Pro polymorphism may contribute to an increased risk of cutaneous melanoma (CM).

**Methods:**

By searching the databases of PubMed, EMBASE, and Web of Science, a total of 8 eligible case-control studies with 1,957 CM cases and 2,887 controls were included in this meta-analysis. Stata software was used to analyze all the statistical data.

**Results:**

The pooled data by a fixed-effects model suggested an increased risk of CM associated with *p53* Arg72Pro polymorphism under the genetic model of Arg/Pro vs. Pro/Pro without heterogeneity (OR_Arg/Pro vs. Pro/Pro_ = 1.76, 95% CI = 1.55-1.99, *P*_heterogeneity_ = 0.075). A similar trend was seen in subgroups of hospital-based studies and population-based studies.

**Conclusion:**

Our meta-analysis based on all studies shows that the *p53* Arg72Pro polymorphism may increase individual susceptibility to CM, particularly in Caucasians and could serve as a biomarker to predict the population at high risk of CM.

## Introduction

Cutaneous melanoma (CM) representing one of the most malignant skin cancers has caused a large number of skin cancer-related deaths, approximately 8,700 deaths in 2010 [1]. The incidence rate steadily rises, with an average increase by 3–7% throughout the past decades among European populations [2]. It has generally been accepted that exposure to ultraviolet (UV) radiation is a main cause of skin cancer [3]. More importantly, epidemiological evidence has documented genetic variations in cancer-related genes are major contributing factors for this malignancy [4,5]. Despite the previous efforts, the pathogeneses of this disease remains unclear.

The tumor suppressor *p53* involved in a variety of biological activities, such as UV-induced DNA damage, regulates numerous downstream genes to induce cell-cycle arrest, DNA repair or apoptosis [6,7]. *p53* is a most frequently mutated gene that has been described in various cancers [8,9] and the single nucleotide polymorphisms (SNPs) at this locus should account, at least in part, for the occurrence of these cancers [10]. Among these, the extensively studied SNP has been the one at codon 72, which has a substitution of Arg to Pro.

Previous reports suggest that *p53* plays a pivotal role in the defence against DNA damage and breakage arsing from UV exposure [11,12]. Due to the key role of *p53* in the development of skin cancer, an increasing number of investigators have directed their attention to the effects of *p53* Arg72Pro polymorphism on CM risk [13–20]. But there is considerable discrepancy in the findings as a result of the small-sampled studies. Therefore, in order to obtain an estimation with more statistical power, we conducted a meta-analysis to test the hypothesis that *p53* Arg72Pro polymorphism may contribute to an increased risk of CM.

## Methods and Materials

### Literature search

We searched the databases of PubMed, EMBASE, and Web of Science for case-control studies on the association between *p53* Arg72Pro polymorphism and CM risk by using the following search terms: “*p53* codon 72” or “*p53* Arg72Pro”, “polymorphism” or “variants”, and “melanoma” or “cutaneous melanoma”. There was no language restriction. References of the original articles and systematic reviews were manually screened for additional usable data.

### Inclusion and exclusion criteria

The inclusion criteria included: (1) the association of *p53* Arg72Pro polymorphism and CM risk must be examined, (2) designed as case-control study, (3) detailed genotype frequency in cases and controls to estimate odds ratios (ORs) along with 95% confidence intervals (CIs), and (4) there was no departure from Hardy-Weinberg equilibrium (HWE) in genotype distribution of the control group. Abstracts, editorials, and review articles were not considered in the final analysis.

### Data extraction

On the basis of a consensus on all items, two reviewers independently collected the following characteristics from each study: first author’s name, publication year, study country, ethnicity, total numbers of genotyped cases and controls, genotype counts of *p53* Arg72Pro polymorphism in cases and controls, and genotyping assays. Disagreement was resolved by discussion between the two reviewers or consulting the third reviewer.

### Statistical analysis

In this meta-analysis, association of *p53* Arg72Pro polymorphism and CM risk was assessed by pooled ORs with 95% CIs under five genetic comparisons (Arg/Arg vs. Pro/Pro, Arg/Arg + Arg/Pro vs. Pro/Pro, Arg/Arg vs. Arg/Pro + Pro/Pro, allele Arg vs. allele Pro, and Arg/Pro vs. Pro/Pro). HWE of the control groups was determined by the χ^2^ test. Between-study heterogeneity was estimated by chi-square based Q test [21] and *P* < 0.05 indicated significant heterogeneity. I^2^ index was also used to quantify the heterogeneity and we considered the value >50% as statistically significant. When no heterogeneity was observed across studies, the fixed-effects model was used [22] to calculate the summary ORs for the combined studies and the random-effects model [23] was performed if there the results were heterogeneous.

In addition, sensitivity analyses were applied to detect the individual influence from the single studies on the pooled ORs. Publication bias was tested by performing Begg’s funnel plots and Egger’s test [24]. All statistical data were analyzed using Stata software (version 12.0, Stata Corp LP, College Station, TX, USA). All tests were two-sided with a significant level of 0.05.

## Results

### Characteristics of the studies

**Fig 1:**
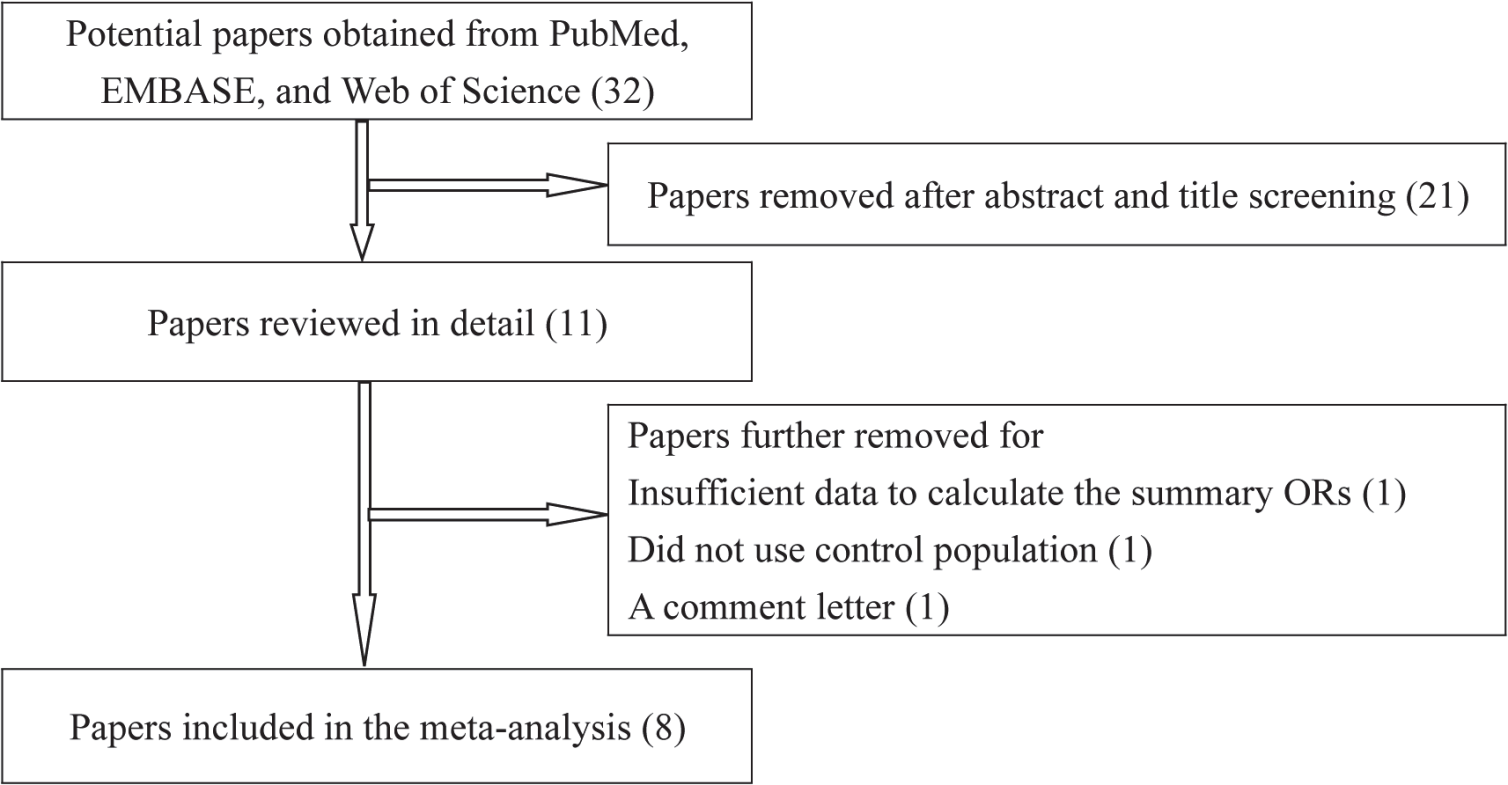
Flow diagram of included studies for this meta-analysis.

As graphically depicted in Fig. 1, the literature search identified 32 potentially relevant articles in all. Of these, 21 papers were excluded by reading the titles and abstracts. After examining the full-texts, we further removed 3 articles due to case-only design [25], insufficient data to calculate the combined ORs [26] and comment letter [27]. At last, a total of 8 case-control studies with 1,957 CM cases and 2,887 controls were pooled in the meta-analysis. Major characteristics of the eligible studies are listed in Table 1. All of the included studies employed Caucasian populations. There were 6 hospital-based studies [13–15,17,19,20] and two population-based studies [16,18]. The numbers of subjects recruited in each of the single studies varied substantially, from the smallest of 239 to the largest of 1,643. No deviation from HWE was seen in the control groups.

**Table 1:** Characteristics of studies included in the meta-analysis.

First author	Year	Country	Ethnicity	Study design	Genotyping method	HWE	Cases	Controls
Bastiaes	2001	Netherland	Caucasian	HB	PCR	0.180	120	157
Shen	2003	USA	Caucasian	HB	PCR-RFLP	0.793	289	308
Gwosdz	2006	Germany	Caucasian	HB	RT-PCR	0.419	49	193
Han	2006	USA	Caucasian	PB	TaqMan	0.864	201	816
Stefanaki	2007	Greece	Caucasian	HB	AS-PCR	0.058	107	145
Li	2008	USA	Caucasian	HB	ND	0.184	805	838
Capasso	2010	Italy	Caucasian	PB	PCR	0.599	240	284
Oliveira	2013	Brazil	Caucasian	HB	PCR	0.757	146	146

Abbreviations: PCR: polymerase chain reaction; PCR-RFLP: PCR-restriction fragment length polymorphism; RT-PCR: real time-PCR; AS-PCR: allele-specific-PCR; TaqMan: TaqManSNP; ND: not defined; HB: hospital-based; PB: population-based; HWE: Hardy-Weinberg equilibrium.

### Meta-analysis results

**Fig 2:**
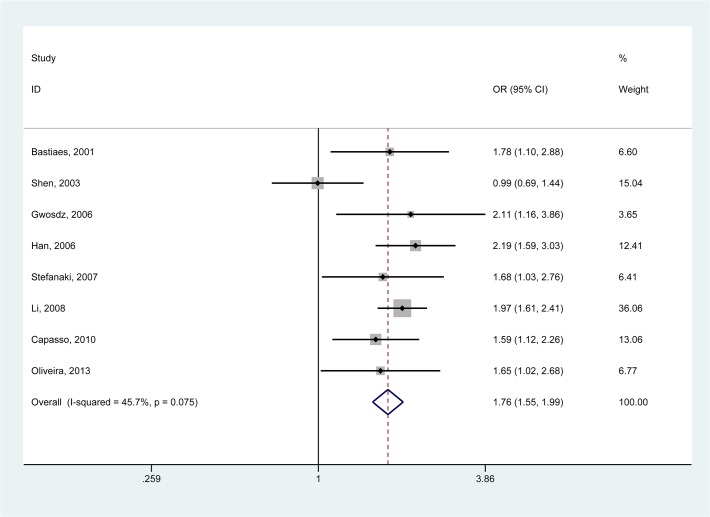
Forest plot (Fixed effects model) describing the association of the *p53* Arg72Pro polymorphism with cutaneous melanoma (CM). The *p53* Arg72Pro polymorphism shows a signficant association with risk of CM (Arg/Pro vs. Pro/Pro).

Since the test for heterogeneity did not suggest substantial heterogeneity across studies, the fixed-effects model was performed to pool the ORs, as shown in Table 2. The combined results showed no statistically significant links between the *p53* Arg72Pro polymorphism and CM susceptibility under all genetic models with the exception of Arg/Pro vs. Pro/Pro (OR_Arg/Pro vs. Pro/Pro_ = 1.76, 95% CI = 1.55–1.99, *P*_heterogeneity_ = 0.075, Fig. 2). When stratifying the populations by source of controls, we observed significantly increased risk in hospital-based studies (OR_Arg/Pro vs. Pro/Pro_ = 1.71, 95% CI = 1.48–1.98, *P*_heterogeneity_ = 0.058) as well as population-based studies (OR_Arg/Pro vs. Pro/Pro_ = 1.88, 95% CI = 1.49–2.39, *P*_heterogeneity_ = 0.186).

**Table 2:** Meta-analysis for the association between the *p53* Arg72Pro polymorphism and CM risk.

Subgroup (cases/controls)	Arg/Arg vs. Pro/Pro		Arg/Arg + Arg/Pro vs. Pro/Pro		Arg/Arg vs. Arg/Pro + Pro/Pro		Allele Arg vs. Allele Pro		Arg/Pro vs. Pro/Pro	
	OR (95% CI)	P_h_	OR (95% CI)	P_h_	OR (95% CI)	P_h_	OR (95% CI)	P_h_	OR (95% CI)	P_h_
Fixed-effects										
Ethnicity										
Caucasian (1957/2887)	1.00 (0.89, 1.12)	0.990	1.00 (0.91, 1.08)	0.999	1.06 (0.96, 1.18)	0.422	1.02 (0.96, 1.09)	0.765	1.76 (1.55, 1.99)	0.075
Control source										
Hospital (1516/1787)	1.02 (0.89, 1.16)	0.970	1.01 (0.91, 1.11)	0.997	1.10 (0.98, 1.24)	0.431	1.04 (0.97, 1.12)	0.709	1.71 (1.48, 1.98)	0.058
Population (441/1100)	0.95 (0.76, 1.18)	0.925	0.97 (0.82, 1.14)	0.869	0.96 (0.79, 1.16)	0.424	0.96 (0.85, 1.09)	0.752	1.88 (1.49, 2.39)	0.186
Total (1957/2887)	1.00 (0.89, 1.12)	0.990	1.00 (0.91, 1.08)	0.999	1.06 (0.96, 1.18)	0.422	1.02 (0.96, 1.09)	0.765	1.76 (1.55, 1.99)	0.075

Abbreviations: Ph: p value of heterogeneity test; CI: confidence interval; OR, odds ratio.

### Sensitivity analyses

We conducted leave-one-out sensitivity analyses with an aim to determine the effects of the independent studies on the overall results. The analysis did not indicate any significant alteration when the single studies was excluded from the pooling data. Thus our results are stable and credible.

### Publication bias

**Fig 3:**
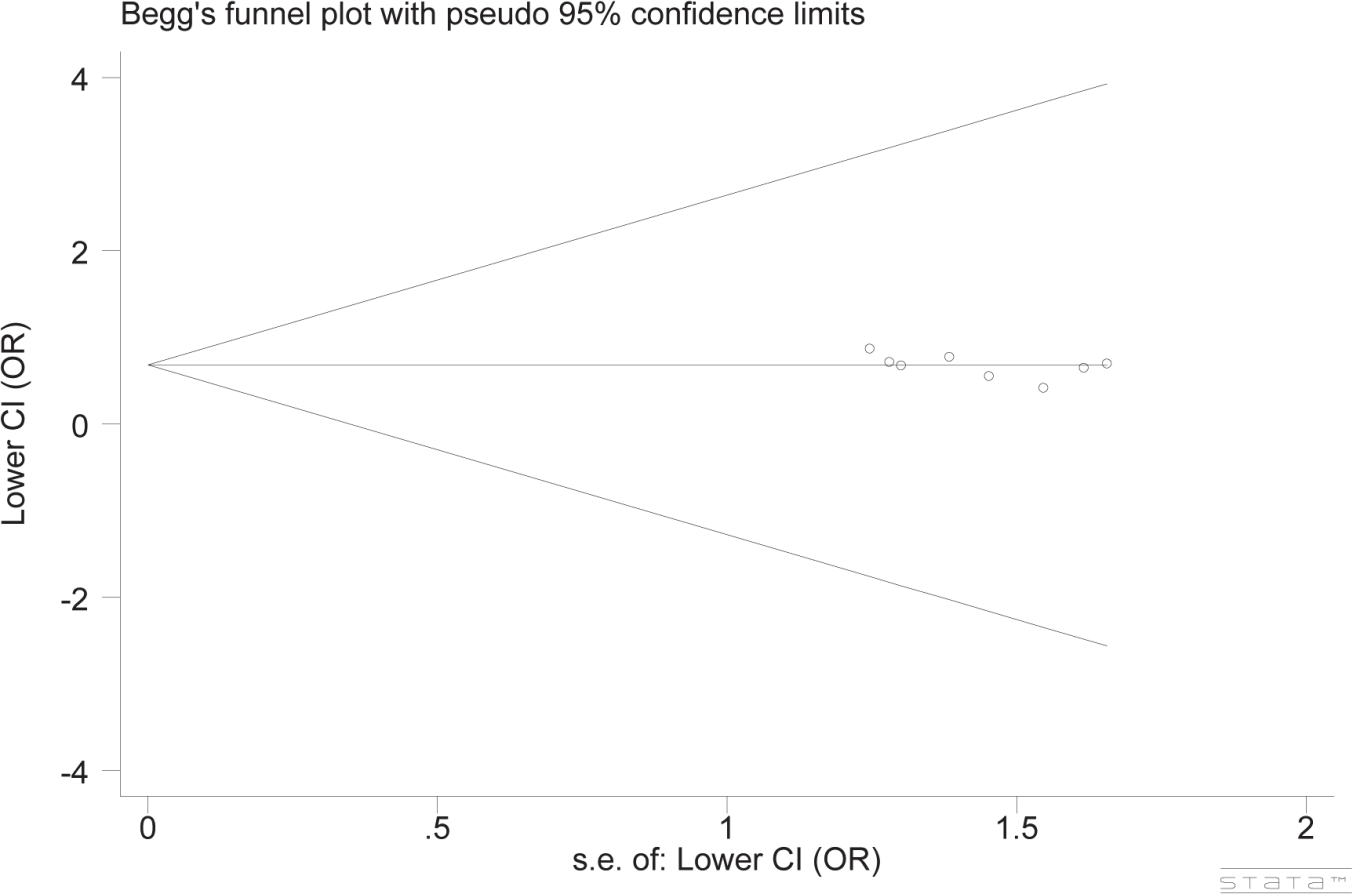
Funnel plot analysis to detect publication bias. Each point represents an individual study for the indicated association. No publication bias was suggested in this meta-analysis (Arg/Arg vs. Pro/Pro).

The results of Begg’s funnel plots and Egger’s test showed that publication bias may not have a significant effect on the findings of our meta-analysis for the association of *p53* Arg72Pro polymorphism and CM susceptibility (Arg/Arg vs. Pro/Pro: *P* = 0.174 for Begg’s test; *P* = 0.134 for Egger’s test, Fig. 3).

## Discussion

In this meta-analysis consisting of 1,957 CM cases and 2,887 controls from 8 eligible case-control studies, we demonstrated that *p53* Arg72Pro polymorphism may contribute to an increased risk of CM. This finding was further confirmed in subgroup analysis by ethnicity, revealing that Caucasians with the Arg/Pro genotype had 1.76-fold higher risk to develop CM compared to the Pro/Pro genotype carriers. In addition, both the studies based on hospital-based controls and those on population-based controls were found to be significantly associated with CM risk. To our knowledge, this is the first study examining the association between *p53* Arg72Pro polymorphism and CM risk in Caucasians. Consistent with our initial hypothesis, our results showed that this polymorphism may have effects on the development of CM.

The *p53* tumor suppressor gene that encodes a DNA-binding protein plays an important role in tumor suppression and cell cycle arrest. Loss of tumor suppression function and cell cycle control resulting from mutations and deletions of the *p53* gene induces a wide range of human malignancies, including CM [28,29]. Several case-control studies have been carried out in an attempt to examine the association between *p53* Arg72Pro polymorphism and CM risk. For example, Bastiaens et al. found no significant association for *p53* Arg72Pro polymorphism and CM risk in a case-control study with 120 cases and 157 controls [13]. Conversely, in another larger case-control study (805 CM patients and 838 heathy controls), Li et al. demonstrated that *p53* Arg72Pro polymorphism contributed to the risk of CM [18]. A plausible explanation for the discrepancy is that the number of subjects between the two studies differs substantially, and it is the small sample that are usually underpowered to derive a precise estimation, leading to biased results as a consequence.

Recently, a meta-analysis investigating the association of *p53* Arg72Pro polymorphism with skin cancer has been published [30]. In this analysis, the authors found the polymorphism of interest was not significantly associated with CM. Neither did the stratified analyses according to ethnicity detect a significant association in any subgroup, a finding that varies substantially from that indicated in our meta-analysis. Although both of the meta-analyses involves Caucasian populations only, our analysis includes three more publications contributing to additional 1,413 unique subjects, which enlarges our study substantially and hence enhances the credibility of our results consequently.

We identified a notably increased risk of CM in carriers of the Arg/Pro heterozygote, a finding that has some biological plausibilities. p53 is a signaling pathway fundamental in tumor growth suppression by promoting cellular proliferation and inducing cell death. Dumont et al. established a linkage of apoptotic potential with a common polymorphism that influences amino acid position72 at p53 locus; the Arg allele has been shown to have greater ability to induce apoptosis most likely due to the close affinity with mitochondria [31]. In addition, the *p53* in conjunction with proopiomelanocortin gene within keratinocytes in defence against UV radiation stimulates melanogenesis, a potent determinant of skin color. The functional *p53* polymorphism modulates proopiomelanocortin activity at the allelic level and thereby confers susceptibility to the development of skin cancer [32]. These data make us infer that the presence of *p53* Arg72Pro genotypes or alleles may possibly affect the function of *p53* and ultimately modifies the risk of skin cancer, providing supportive evidence for an association between the *p53* Arg72Pro and CM. Some limitations in our meta-analysis need to be addressed. To begin with, this meta-analysis is based on Caucasians only and reveals significantly increased risk of CM ascribed to the *p53* Arg72Pro polymorphism. However, we can not exclude the possibility that the contribution of the polymorphism to the risk of CM differs due to different ethnic origins, and it may not represent a risk factor for other ethnicities. Furthermore, since only English-language and published articles are included in our study, selection bias may have occurred. Finally, there are no uniform criteria defined for the selection of control subjects in each of the studies included, some used population-based controls and the others selected hospital-based controls, so potential selection bias might exist in this study.

In summary, our meta-analysis provided some evidence that the Arg/Pro genotype of *p53* Arg72Pro polymorphism was likely to confer susceptibility to CM. Future larger studies with the consideration of more ethnic groups and representative control groups are necessary to further identify our findings.

## Supporting Information

S1 PRISMA Checklist (DOC)
